# Supplementary material for: The Pro-Gly or Hyp-Gly Containing Peptides from Absorbates of Fish Skin Collagen Hydrolysates Inhibit Platelet Aggregation and Target P2Y12 Receptor by Molecular Docking
Source: Foods. 2021 Jul 5;10(7):1553. doi: 10.3390/foods10071553 (PMC8303285; doi:10.3390/foods10071553)
Supplement: Supplementary file 1 [file foods-10-01553-s001.zip › foods-1253985-supplementary.pdf]

Supplementary materials

Figure S1. Inhibition rates and IC_{50} values of 11 identified peptides on platelet aggregation *in vitro*.

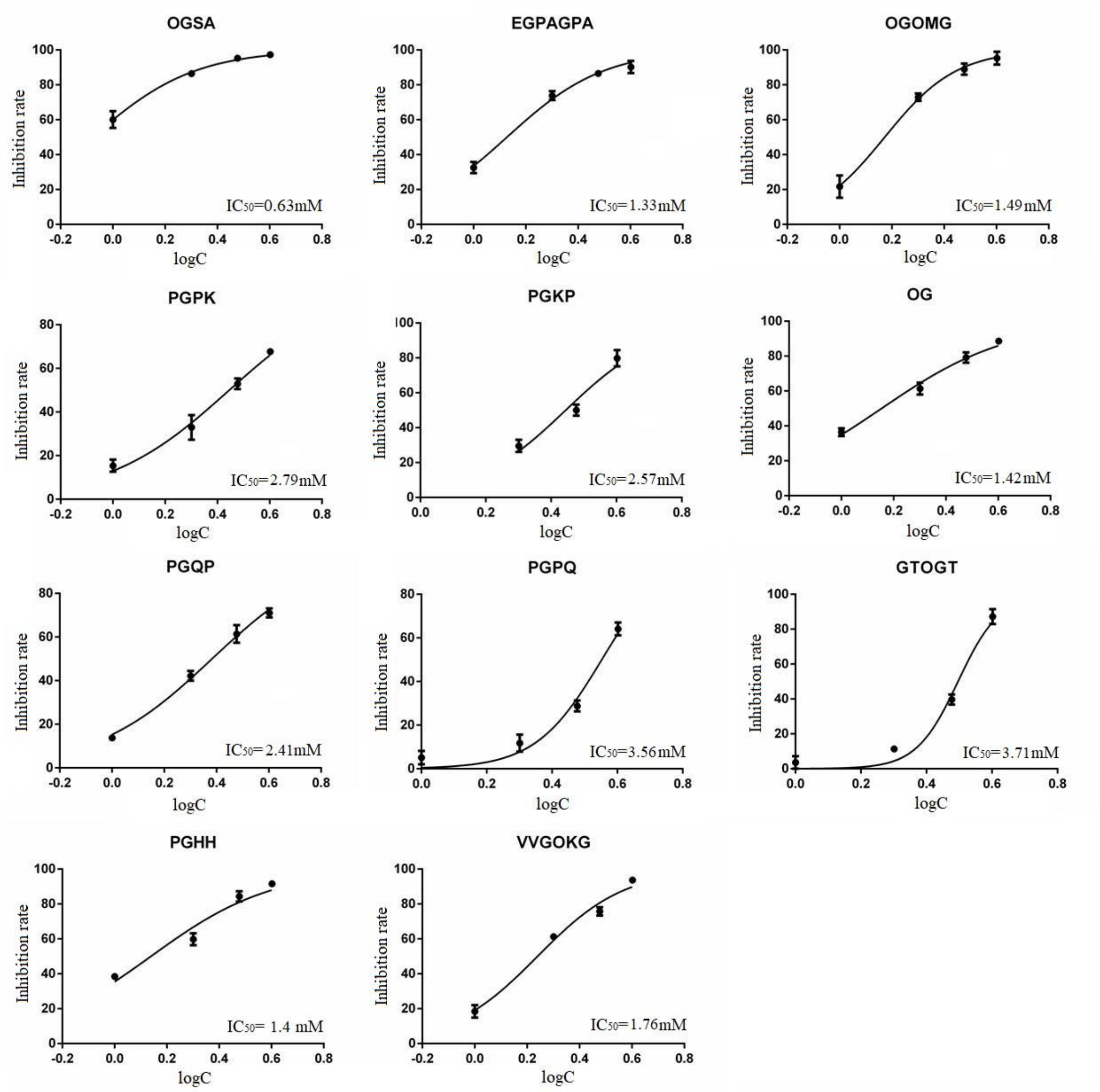

Figure S2. The effect of different doses of OGSA and Clopidogrel on thymus index and spleen index *in vivo*. Mean ± S.D, n=5.

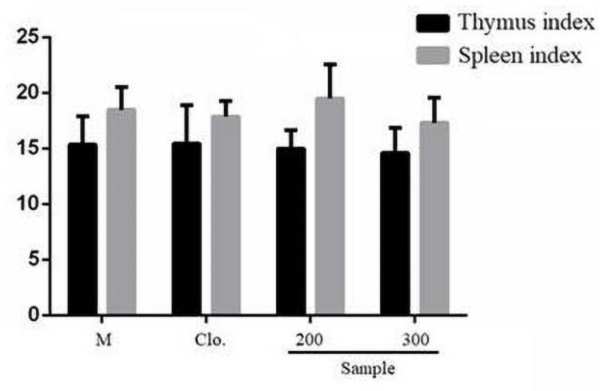

Figure S3. Amino acid sequence of type I collagen of Hypophthalmichthys Molitrix skin.

mfsfvdirlalllsatvllargqgeddrtggsctldgqvyndrdvwkpepcqicvcdsgtvmcdevicedttdcpnpviphdeccpvcpdddfqepsv
egprgtpgekgdrgppgppgndgipgqpglpgppgppgppglggnfspqmsggfdeksggamavpgpmgpmgprgppgppgtpgpqgftg
ppgepgeagapgpmgprgaagppgkngedgesgkpgrpgergppgpqgargfpgtpglpgikghrgfsgldgakgdtgpsgpkgeagapgengt
pgamgprglpgergragppgaagargndgaagaagppgptgpagppgfpggpgakgevgpqgargaegpqgargeagnpgpagpagpagnng
adgaagpkgspgtpgiagapgfpgprgppgpsgaagapgpkgntgevgapgakgeagakgeagaqgvqgppgppgeegkrgargepgaaggrg
ppgergapgargfpgadgsagpkgapgerggpgvvgpkgatgepgrngepgmpgskgmtgspgspgpdgktgpsgtpgqdgrpgppgpvgar
gqpgvmgfpgpkgaageagkpgergvmgavgatgapgkdgdvgapgapgpagpagergeqgpagppgfqglpgpqgatgepgksgeqglpg
eagapgpsgsrgdrgfpgergapgpagpagargspgsagndgakgdsgapgapgaqgppglqgmpgergaaglpglkgdrgdqgakgtdgapgk
dgirgmtgpigppgpagapgdkgetgapglvgpagargppgergetgapgpagfagppgadglpgakgeagdngakgdagppgpsgatgapgpq
gpvgatgpkgargaagppgatgfpgaagrvgppgpagnagppgppgpagkegqkgsrgetgpagrtgeigtpgppgapgekgtpgaegptgpsgt
pgpqgingqrgivglpgqrgergfpglpgpsgepgkqgpsgpsgergppgpmgppglagppgepgregtpgnegsagrdgapgpkgdrgetgaa
gtgapgpigpagktgdrgesgpagpsgavgltgprgpvgpagargdkgetgeagergmkghrgftgiqgppgppgpsgepgpagasgpagprgpa
gssgpagkdgmsglpgpigppgprgrngeigpagppgapgppgppgpsgggfdigfiaqpqekapdpfrhfraddanvmrdrdlevdttlkslsqq
iesimspdgtkknpartcrdlkmchpdwksgeywidpdqgcnqdaikvycnmetgetcvyptestipkknwytsknikekkhvwfgeamtdgfq
feygsegskaedvniqltflrlmsteasqnityhcknsiaymdqasgnlkkalllqgsneieiraegnsrftysvtedgctshtgawgktvidykttktsrlp
iidiapmdvgapnqefgievgpvcfl

Table S1. Molecular docking scores of 13 antiplatelet peptides with P_2Y_{12} receptor.

Peptide	Crash	Polar	Similarity	D Score	PMF Score	G Score	Chem Score	C Score	Total Score
OGSA	-1.46	8.09	0.42	-191.18	-112.58	-288.29	-18.33	4	11.94
PGE	-2.23	6.38	0.50	-176.11	-77.71	-299.98	-17.57	3	11.43
GTOGT	-7.11	9.61	0.46	-235.80	-128.21	-422.96	-20.29	3	10.32
OGOMG	-7.75	7.13	0.57	-258.63	-72.96	-457.65	-27.03	3	10.14
PGPK	-4.93	4.40	0.46	-144.04	-92.79	-389.00	-29.17	3	9.66
PGPQ	-5.33	5.27	0.50	-182.52	-106.21	-346.01	-17.33	3	9.38
PGEOG	-6.79	6.24	0.54	-288.35	-106.85	-478.71	-13.53	4	9.03
OGE	-3.42	7.03	0.50	-213.38	-56.12	-284.88	-16.55	3	8.87
PGHH	-7.13	5.33	0.52	-224.26	-137.47	-383.18	-24.67	4	8.26
PGKP	-7.55	2.88	0.47	-163.59	-35.03	-405.74	-20.63	1	7.11
OG	-1.28	3.71	0.37	-113.05	-34.87	-177.12	-9.89	3	6.92
PGQP	-5.84	4.19	0.54	-196.94	-75.67	-338.86	-18.73	3	6.49
VGPOGPA	-17.12	7.82	0.52	-307.32	-109.42	-572.97	-40.54	3	5.14

Table S2. The sites of identified peptides in skin collagen of *Hypophthalmichthys molitrix*.

Sample	No.	M	Peptide	Sites [a]
A1	1	598.25	EGPAGPA	α2(I) 455-461
	2	447.72	GTO(P)GT	α2(I) 198-202
	3	398.21	PGPK	α1(I) 412-415, 568-571, 1015-1018; α2(I) 251-254, 485-488, 641-644
	4	398.21	PGPQ	α1(I) 181-184, 232-235, 631-634, 835-838, 934-937; α2(I) 98-101, 320-323
	5	398.21	PGQP	α1(I) 123-126
	6	398.21	PGKP	α2(I) 45-48, 140-143, 494-497
	7	347.15	O(P)GSA	α1(I) 645-648; α2(I) 353-356
	8	189.12	O(P)G	Fragments=117 in α1(I) and 99 in α2(I)
A4	9	573.63	VVGO(P)KG	α2(I) 501-506

	10	490.78	O(P)GO(P)MG	α1(I) 163-167, 199-204, 982-986; α2(I) 80-84
P3	11	470.24	P(P)GHH	α2(I) 1067-1070

[a] The sites information comes from the α1(I) chain and α2(I) chain of type I collagen of *Hypophthalmichthys molitrix* in the NCBI database.

(https://www.ncbi.nlm.nih.gov/protein/AIL02135.1; https://www.ncbi.nlm.nih.gov/protein/AUF74474.1).
